# Comparative Transcriptome Analysis Revealed Key Genes Regulating Gossypol Synthesis in Tetraploid Cultivated Cotton

**DOI:** 10.3390/genes14061144

**Published:** 2023-05-24

**Authors:** Linglei Kong, Shaoqi Li, Yuyuan Qian, Hailiang Cheng, Youping Zhang, Dongyun Zuo, Limin Lv, Qiaolian Wang, Junlan Li, Guoli Song

**Affiliations:** 1 State Key Laboratory of Cotton Biology, Institute of Cotton Research, Chinese Academy of Agricultural Sciences, Anyang 455000, China; linglei_kong@foxmail.com (L.K.); pser2010@163.com (H.C.); zyp547550790@163.com (Y.Z.); zdy041@163.com (D.Z.); llm0372@126.com (L.L.); wangqiaolian@caas.cn (Q.W.); 2 Semi-Arid Agriculture Engineering & Technology Research Center of P. R. China, Shijiazhuang 050051, China; 3 Institute of Cotton, Hebei Academy of Agriculture and Forestry Sciences, Key Laboratory of Cotton Biology and Genetic Breeding in Huanghuaihai Semiarid Area, Ministry of Agriculture and Rural Affairs, National Cotton Improvement Center Hebei Branch, Shijiazhuang 050051, China; li_shaoqi@foxmail.com (S.L.);

**Keywords:** tetraploid cultivated cotton, pigment gland, gossypol, transcriptome, WGCNA

## Abstract

Tetraploid cultivated cotton (*Gossypium* spp.) produces cottonseeds rich in protein and oil. Gossypol and related terpenoids, stored in the pigment glands of cottonseeds, are toxic to human beings and monogastric animals. However, a comprehensive understanding of the genetic basis of gossypol and gland formation is still lacking. We performed a comprehensive transcriptome analysis of four glanded versus two glandless tetraploid cultivars distributed in *Gossypium hirsutum* and *Gossypium barbadense*. A weighted gene co-expression network analysis (WGCNA) based on 431 common differentially expressed genes (DEGs) uncovered a candidate module that was strongly associated with the reduction in or disappearance of gossypol and pigment glands. Further, the co-expression network helped us to focus on 29 hub genes, which played key roles in the regulation of related genes in the candidate module. The present study contributes to our understanding of the genetic basis of gossypol and gland formation and serves as a rich potential source for breeding cotton cultivars with gossypol-rich plants and gossypol-free cottonseed, which is beneficial for improving food safety, environmental protection, and economic gains of tetraploid cultivated cotton.

## 1. Introduction

Tetraploid cultivated cotton (*Gossypium* spp.) is a globally appreciated and important economic crop, which produces the leading natural fiber for textile and provides a large quantity of cottonseeds containing 21% oil and 23% protein [1]. However, the oil or protein from cottonseeds is inedible directly due to the presence of gossypol, which is toxic to humans and monogastric animals [2,3]. It must be dephenolized before application, which limits the comprehensive utilization of cottonseed. Gossypol is a yellowish phenolic compound that can protect cotton plants against pests, diseases, and abiotic stresses [4,5]. Therefore, breeders have exerted much effort to cultivate cotton varieties with gossypol-free cottonseed and plants with a normal gossypol content [1,6,7].

Pigment glands appear as small dark spots and are specialized cavity structures that store a wide variety of secondary metabolites, including gossypol [8,9]. Given the specific storage locations of gossypol and related terpenoids, they are significant indicators of gossypol, with significant positive correlations with the numbers [10,11]. Therefore, it is important to explore the genetic basis of pigment glands for developing cotton cultivars with gossypol-free cottonseed and plants with a normal gossypol content. Pigment glands are one of the major characteristics of the *Gossypium* species [12]. They are secretory organs with a large central cavity which are formed by the degradation of gland primordium cells [13]. Most of the pigment glands are distributed in the subepidermal tissues of the aerial parts and the cortex of roots in cotton plants [3].

Studies on the genetic mechanisms of pigment glands formation in cotton began in the early 20th century [14,15,16,17]. Since then, genetic research conducted by cotton breeders revealed the roles of six gland genes in gland formation, of which two major genes were *Gl_2_* and *Gl_3_* [18]. In a previous study, the glandless trait of the whole plant and cottonseed was controlled by two recessive genes (*gl_2_gl_2_gl_3_gl_3_*) on A12 and D12 chromosomes or one dominant gene (*Gl_2_^e^*) at the *Gl_2_* locus [19,20,21]. The *Gl_2_^e^* was discovered in a mutant released as Bahtim 110 in Egypt and Hai 1 (*G. barbadense*) in China [21,22]. Then, it was identified through map-based cloning [23,24]. The *gl_1_* was mapped based on bulked segregant analysis and sequencing [25]. Virus-induced gene silencing (VIGS) of the candidate *GoSPGF* resulted in glandless stems and a dramatically reduced gossypol content [25]. In addition, three ‘Cotton Gland Formation’ genes (*CGF1*, *CGF2,* and *CGF3*) were identified using the RNA-seq analysis of embryos from near-isogenic glanded (*Gl_2_Gl_2_Gl_3_Gl_3_*) versus glandless (*gl_2_gl_2_gl_3_gl_3_*), and VIGS against them led to significant reductions in the gland number in the leaves and a significantly lower gossypol level and related terpenoids [2]. A new gland-associated gene *GauGRAS1* was identified in the gland-forming stage and functionally validated by VIGS, which caused glandless stems and petioles in *Gossypium australe* [26]. A transcription factor named *CGP1* was identified by comparative transcriptome analysis on the stem tissues of glanded and glandless varieties, which was involved in regulating gland pigmentation [3]. Although several genes related to gland formation or gossypol synthesis have been discovered and cloned, the regulatory relationships between them are still not clear. Understanding the specific mechanism of pigment gland formation and gossypol synthesis could facilitate the cultivation of glandless cottonseeds without affecting the amount of gossypol in the whole plant.

Recently, weighted gene co-expression network analysis (WGCNA) has been widely applied as a powerful method to illustrate the genetic architecture underlying complex traits by extracting meaningful differences across the integration of large-scale transcripts and complex traits [27,28,29,30]. The gene sets and their co-expression modules, including hub genes which play a key role in the regulation of the gene expression network, can be identified effectively, which are strongly specifically associated with the target traits [31]. WGCNA has been widely used to study fiber traits [32,33,34], stress resistance [35], and so on, but few studies focused on gossypol and pigment glands exist.

In this study, we employed comparative transcriptome and WGCNA analysis of glanded and glandless cultivars distributed in *G. hirsutum* and *G. barbadense* to identify the major genes involved in gland formation in tetraploid cultivated cotton. A series of differentially expressed genes were identified in the leaves from glanded and glandless cotton cultivars. Moreover, a WGCNA analysis was performed using differentially expressed genes to identify important co-expression modules and related metabolic networks, which were strongly associated with the formation of the pigment gland and related secondary metabolism. Finally, the hub genes were highlighted and further validated using a quantitative reverse transcription polymerase chain reaction (qRT-PCR). The results of this study provide insight into the molecular genetic basis of gossypol gland formation and serve as a rich potential source for developing cotton cultivars with gossypol-free cottonseed and plants with a normal gossypol content.

## 2. Materials and Methods

### 2.1. Plant Materials

Four glanded cultivars (*G. hirsutum* ‘X10’, *G. hirsutum* ‘TM–1’, *G. barbadense* ‘H7124’, and *G. barbadense* ‘3–79’) and two glandless cultivars (*G. hirsutum* ‘X18’ and *G. barbadense* ‘H1’) obtained from the Institute of Cotton Research, Chinese Academy of Agricultural Sciences (Anyang, China) were planted at Anyang Experiment Station. Among these cultivars, ‘X10’, ‘TM–1’, ‘H7124’, and ‘3–79’ have glands throughout the whole plant and cottonseed. ‘H1’ is produced by introducing *Gl_2_^e^* in *G. barbadense* and shows no gland in the whole plant and cottonseed [21]. X18 is a mutant bred by dominant glandless line Zhong 5655 (*G. hirsutum*, bred from H1) with backcrossing glanded line X10 through years of selections [36,37,38]. X18 has the special property of producing low-gossypol cottonseed and has a few glands in its stem and vein, but no glands in its leaves. Apical fresh leaves of each cultivar were collected without veins at the full-bloom stage (approximately week 12 after emergence) for total RNA extraction. Three biological replicates were maintained for each cultivar and frozen in liquid nitrogen immediately and stored at −80 °C.

### 2.2. RNA Extraction, Library Construction, and RNA Sequencing

Total RNA was isolated from the collected leaves of each sample using the Plant RNA Rapid Extraction kit (Molfarming, Nanjing, China). RNA quality and concentration were examined with the Agilent 2100 RNA 6000 Nano kit (Agilent Technologies, Santa Clara, CA, USA). Only the RNA samples with OD260/280 = 1.8–2.2, OD260/230 > 2.0 and RNA integrity number (RIN) > 8 were used for RNA sequencing. Library construction and sequencing were accomplished by Majorbio Bio-pharm Technology Corporation, Ltd. (Shanghai, China). The mRNA was enriched from total RNA using magnetic beads with oligo (dT) for library preparation. A total of 18 libraries were sequenced using the Illumina Novaseq 6000 platform with 2 × 150 bp paired-end raw reads.

### 2.3. RNA-seq Data Analysis

The RNA-seq raw data were processed to filter out the adapter, poly-N, and low-quality reads using Trimmomatic (v0.36) software [39]. The clean data were mapped to the reference genome of TM–1 (*G. hirsutum*) [40] using HISAT2 [41]. Gene expression values were estimated using the Subread suite (v1.5.2) [42], and the transcripts per kilo-base of exon model per million mapped reads (TPM) were calculated to measure the gene expression level [43]. Pearson correlation coefficients between samples were calculated, and samples with correlation coefficients less than 0.8 between the biological replicates were eliminated. The differentially expressed genes (DEGs) between glandless and glanded samples were identified based on the average expression of biological replicates using DESeq2 R package [44], which were carried out in *G. hirsutum* and *G. barbadense* samples, respectively. The genes with padj ≤ 0.05 and an absolute value of log2 fold change ≥ 1.5 were defined as significant DEGs. A Venn diagram and volcano plot were drawn using the R package ggplot2 [45].

### 2.4. Construction of Gene Co-Expression Networks

Gene co-expression networks were constructed using the R pipeline WGCNA [31]. The common DEGs were clustered into co-expression modules, and the correlations between each module eigengene and glanded/glandless trait were used to estimate module–trait associations. When the co-expression analysis was completed, the edge files were sorted according to the weight value. The co-expression networks among DEGs in the candidate modules that significantly related to the glanded/glandless trait were established with an eigengene-based connectivity (*K_ME_*) value ≥ 0.9 and edge weight value ≥ 0.5. Network visualization was performed using the Cytoscape software version 3.6.1 [46]. Furthermore, hub genes, which show the most significant connections in networks, were identified on the basis of their high module membership (*K_ME_*) values > 0.97 and gene significance > 0.55.

### 2.5. Function Annotation of DEGs

The function annotation of DEGs within the candidate module was performed according to their homology with the annotated *Arabidopsis* genes from TAIR (*Arabidopsis* information resources). Gene Ontology (GO) functional enrichment and Kyoto Encyclopedia of Genes and Genomes (KEGG) pathway analysis were performed on the Cotton Functional Genomics Database (CottonFGD: http://cottonfgd.org/ (accessed on 8 October 2021)) [47] with the criterion of corrected *p*_Value_ ≤ 0.05.

### 2.6. Validation of DEGs by qRT-PCR

First-strand cDNA was synthesized from the total RNA of each sample using the HiScript II Reverse Transcriptase Kit (Vazyme, Nanjing, China). The cDNA was diluted to 100 ng/µL and mixed with TransStart TOP Green qPCR SuperMix (TransGen, Beijing, China) to a total of 20 µL for qRT-PCR. The amplifications were conducted on an ABI Prism 7500 Fast Real-time PCR System (Applied Biosystems, Foster City, CA, USA) according to the instructions provided by the manufacturer. Each qRT-PCR reaction included three biological replicates and three technical replicates. The expressions levels were normalized using *ACTIN* (GenBank: *AY305733*) as an internal reference and calculated using the 2^−∆∆Ct^ method [48]. The specific primers (Table S6) were designed using Oligo 7 software [49] and synthesized by Sangon Biotech (Shanghai, China).

## 3. Results

### 3.1. Transcriptome Analysis

To explore the molecular basis underlying pigment gland formation, we compared the transcriptomes of four glanded and two glandless cultivars distributed in *G. hirsutum* and *G. barbadense*. In total, 18 RNA-seq libraries each with 6 cultivars with 3 biological replications were constructed, and a total of 124.76 Gb clean data from 836.44 million clean reads were generated. The clean data of each sample were more than 6.22 Gb, with a quality score Q30 > 92.59%, and the average GC content was 44.57%. The clean reads of each sample were mapped to the *G. hirsutum* reference genome of TM–1 [40], and the alignment rate ranged from 95.82% to 97.83% (Table S1).

**Figure 1:**
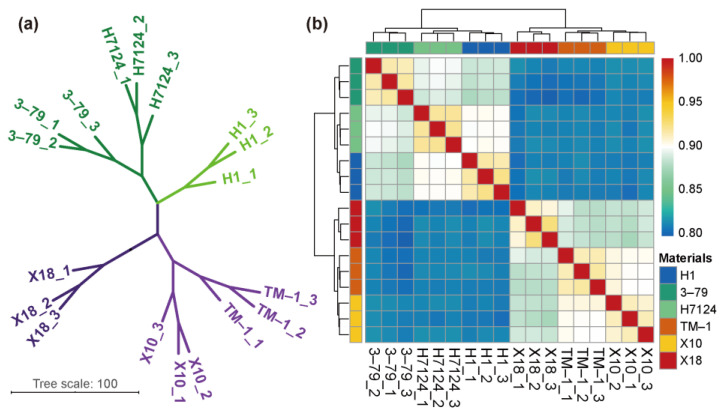
Correlation analysis between samples. (**a**) Principal component analysis (PCA) of genes identified from six cultivars with three biological replicates of each. (**b**) Correlation analysis between 18 samples.

To further exploit RNA-seq results, we employed correlation analysis for each sample based on the expression of all genes, which showed that the *G. hirsutum* and *G. barbadense* samples as well as the glanded and glandless samples were significantly divided into different groups (Figure 1).

### 3.2. Identification of DEGs

**Figure 2:**
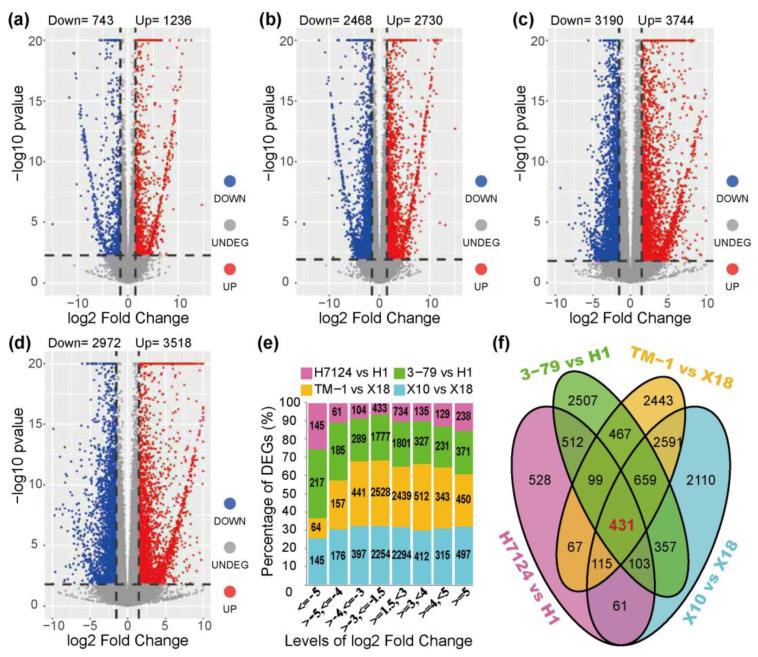
Identification of differentially expressed genes. (**a**–**d**) Volcano map of the differentially expressed genes between cultivars: (**a**) H7124 vs. H1, (**b**) 3–79 vs. H1, (**c**) TM–1 vs. X18, (**d**) X10 vs. X18. (**e**) The distribution of the log_2_ (fold change) of DEGs. (**f**) The statistics of DEGs among the groups for glanded vs. glandless materials.

The DEGs were identified independently across genetic backgrounds due to the extremely significant separation of gene expressions between *G. hirsutum* and *G. barbadense* cultivars (Figure 1). In the group of *G. barbadense* cultivars, a total of 1979 and 5198 DEGs were identified in the pairs of “H7124 vs. H1” and “3–79 vs. H1”, respectively (Figure 2a,b and Table S7). In “H7124 vs. H1”, 1236 (62.46%) DEGs were up-regulated while 743 (37.54%) DEGs were down-regulated (Figure 2a). In “3–79 vs. H1”, 2730 (52.52%), the DEGs were up-regulated while 2468 (47.48%) DEGs were down-regulated (Figure 2b). In the group of *G. hirsutum* cultivars, a total of 6490 and 6934 DEGs were identified in the pairs of “X10 vs. X18” and “TM–1 vs. X18”, respectively (Figure 2c,d and Table S7). In “TM–1 vs. X18”, 3744 (53.99%) DEGs were up-regulated while 3190 (46.01%) DEGs were down-regulated (Figure 2c). In “X10 vs. X18”, 3518 (54.21%) DEGs were up-regulated while 2972 (45.79%) DEGs were down-regulated (Figure 2d). 

A total of 13,113 unique DEGs were identified in the 4 pairs of glanded and glandless comparisons. Of the up-regulated genes (glanded vs. glandless), 7268 (64.73%) had a 1.5–3-folds change in expression, while 1556 (13.86%) had an over 5-folds change in gene expression. Among the down-regulated genes, 6992 (74.60%) had a 1.5–3-folds expression difference, while 571 (6.09%) had a >5-folds expression difference (Figure 2e). Notably, 3858 DEGs were shared between the pair-wise comparisons of “X10 vs. X18” and “TM–1 vs. X18”, 1208 DEGs were shared between the pair-wise comparisons of “3–79 vs. H1” and “H7124 vs. H1”, and 431 common DEGs were shared between the 3858 DEGs of the *G. hirsutum* group and the 1208 DEGs of the *G. barbadense* group (Figure 2f).

### 3.3. WGCNA Analysis of DEGs

**Figure 3:**
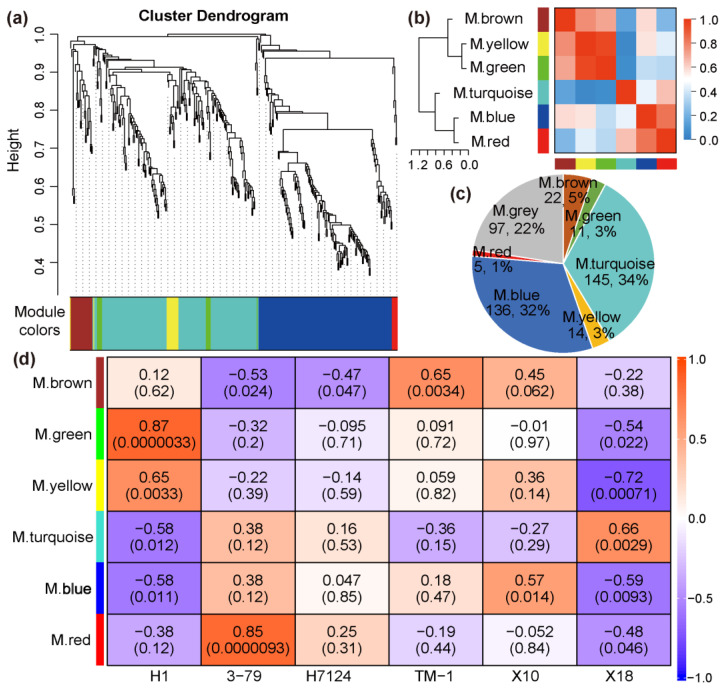
The clustering and module division of DEGs. (**a**) Hierarchical clustering tree showing co-expression modules. (**b**) Correlation analysis between modules. (**c**) Distribution of gene number in co-expression modules. (**d**) The weight correlation between modules and traits (cultivars).

In order to identify the specific gene sets that are strongly correlated with pigment gland formation, the co-expression modules were generated by WGCNA using the TPM of 431 DEGs of all samples. The power of β = 18 (R^2^ = 0.84) was selected as a soft threshold to ensure a scale-free network. Some genes with a higher correlation coefficient were clustered into the same cluster, and then the dynamic cutting method was used to cut the branches into different modules and the modules with similar expression patterns were merged according to a correlation coefficient greater than 0.8. As a result, a total of seven distinct modules associated with the specific expression profiles of different samples were obtained (Figure 3a,b). The turquoise module contains the largest number of genes, and the red module contains the least number of genes (Figure 3c). The grey module represents genes which cannot be classified into any one module and/or whose TPM < 1 in more than 50% of the samples.

**Figure 4:**
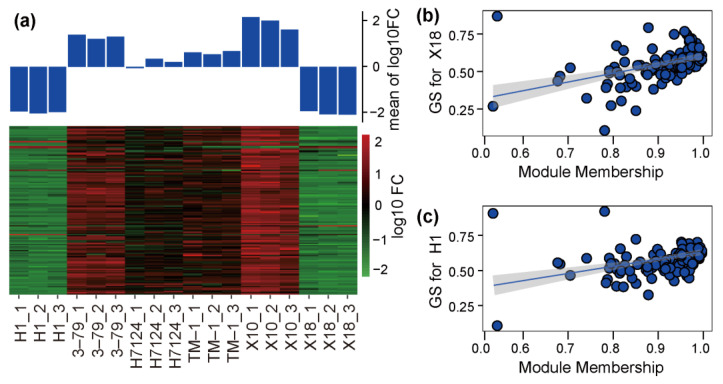
Expression and significance of genes in candidate module. (**a**) The expression heatmap of genes in each sample. (**b**) A scatterplot of gene significance (GS) for X18 vs. Module membership (MM) in blue module. (**c**) A scatterplot of GS for H1 vs. MM in blue module.

The correlation coefficients between trait features of each sample (glanded/glandless) and module eigengenes are shown in Figure 3d. No module is significantly associated with the glanded trait features of H7124, 3–79, TM1, and X18 at the same time. The module eigengenes of ‘green’ and ‘yellow’ were extremely significantly positively associated with the glandless trait features of H1 but significantly negatively associated with the glandless trait features of X18. On the contrary, the module eigengene of ‘turquoise’ was extremely significantly positively associated with the glandless trait features of X18 but significantly negatively associated with the glandless trait features of H1. Of particular note, the module eigengene of ‘blue’, which included 136 genes, was significantly negatively associated with the glandless trait features of both H1 and X18 and positively associated with the glanded trait features of H7124, 3–79, TM1, and X18 at the same time, which showed a consistent association between *G. hirsutum* and *G. barbadense* cultivars (Figure 3d). Consequently, the ‘blue’ module was selected as a candidate module, which could be used to identify the common genetic basis for gland formation in tetraploid cultivated cotton. Corresponding to the significant negative association of the glandless and ‘blue’ module, the expression of most genes in this module was significantly down-regulated in H1 and X18 (Figure 4a). Moreover, significant positive correlations were observed between the gene significance (GS, or the correlation of gene expression and trait features values) and module membership (or *K_ME_*) of each gene in H1 and X18 (Figure 4b,c). In addition, the 136 genes of the ‘blue’ module were spread over the genome, except for chromosome A02, and a pair of homologous chromosomes, A01 and D01, carried the most genes (Table S2).

### 3.4. Construction of Co-Expression Gene Networks and Identification of Hub Genes

**Figure 5:**
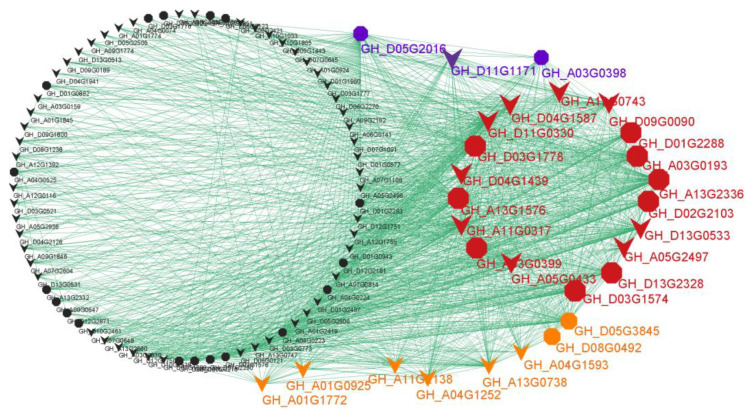
The co-expression network of genes in blue module. Red: common hub genes for H1 and X18; blue: unique hub genes for X18; orange: unique hub genes for H1; black: non-hub genes. Octagon: the homologous genes of known genes related to the gossypol and/or glands formation; V-shaped: the genes that have not been reported.

The WGCNA analysis helped us to focus on the ‘blue’ module, in which a completeness co-expression network was constructed (Figure 5 and Table S2). Interestingly, 21 hub genes were identified based on the criteria of *K_ME_* ≥ 0.98 and gene significance for X18 ≥ 0.57 (Figure 4b and Figure 5 (red and blue)), and 26 hub genes were identified based on the criteria of *K_ME_* ≥ 0.98 and gene significance for H1 ≥ 0.55 (Figure 4c and Figure 5 (red and orange)). There were 18 common hub genes which showed the strongest co-expression correlations. Additionally, a total of 42 genes in the network were identified as homologous genes of the known genes related to the formation of gossypol and glands, and 13 of these were hub genes (Figure 5 (octagons) and Table S2).

### 3.5. Functional Annotations of DEGs in Candidate Module

To identify and confirm the roles of the candidate ‘blue’ module in the formation of gossypol and glands, the DEGs were annotated using functional annotation, GO enrichment, and KEGG pathway enrichment. The results of the functional annotation showed that 123 genes were accurately annotated based on the high homology with *Arabidopsis thaliana* (Table S3). Notably, 24 genes were annotated as ‘cytochrome P450 (CYP) family’, among which 9, 7, and 7 genes were *CYP71B*, *CYP706A,* and *CYP82C*, respectively. Moreover, nine genes were annotated as ‘NAD(P)-binding superfamily protein’ involved in oxidation reduction process; seven genes were annotated as ‘terpene synthase/cyclases’; and five genes were annotated to each of ‘disease resistance responsive family protein’, ‘lactoyl-glutathione lyase (glyoxalase I) family protein’, and ‘transmembrane protein’, respectively. Additionally, 24 of the 29 hub genes were accurately annotated, which were also mainly related to ‘cytochrome P450 (CYP) family’, ‘NAD(P)-binding superfamily protein’, ‘lactoyl-glutathione lyase (glyoxalase I) family protein’, ‘terpene synthase/cyclases’, and ‘2-oxoglutarate (2OG) and Fe(II)-dependent oxygenase superfamily protein’ (Table S3).

**Figure 6:**
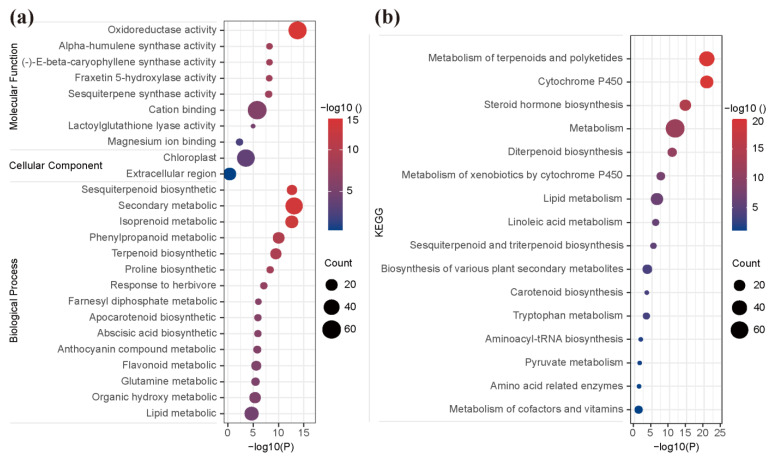
GO and KEGG enrichment. Statistics of GO (**a**) and KEGG (**b**) enrichment of DEGs in blue module. The dot size represents the number of genes, and dot color represents the *p*-value.

The results of GO enrichment showed that 136 DEGs were significantly enriched in 48, 2, and 12 terms of biological processes, cellular components, and molecular functions, respectively (Table S4 and Figure 6a). In the biological process category, the unigenes were prominently enriched in sesquiterpenoid biosynthetic (GO:0016106), secondary metabolic (GO:0019748), isoprenoid metabolic (GO:0006720), phenylpropanoid metabolic (GO:0009698), and terpenoid biosynthetic (GO:0016114) (Figure 6a). In the cellular component category, the unigenes were significantly enriched in the chloroplast (GO:0009507) and extracellular region (GO:0005576) (Figure 6a and Table S4). In the molecular function category, the unigenes were prominently enriched in oxidoreductase activity (GO:0016491), alpha-humulene synthase activity (GO:0080017), (-)-E-beta-caryophyllene synthase activity (GO:0080016), fraxetin 5-hydroxylase activity (GO:0106144), sesquiterpene synthase activity (GO:0010334), and cation binding (GO:0043169) (Figure 6a). Moreover, most of the hub genes were mainly enriched in the five biological process categories, which were secondary metabolic (GO:0019748), isoprenoid metabolic (GO:0006720), terpenoid biosynthetic (GO:0016114), organic hydroxy metabolic (GO:1901615), and lipid metabolic (GO:0006629). Of particular note, five hub genes, *GH_D09G0090*, *GH_D01G2288*, *GH_A13G1576*, *GH_D05G3845,* and *GH_A01G0925,* were significantly enriched in more than sixteen terms of GO enrichments, especially in sesquiterpene synthase activity (GO:0010334), sesquiterpenoid biosynthetic (GO:0016106), isoprenoid metabolic (GO:0006720), and terpenoid biosynthetic (GO:0016114), which were directly involved in gossypol biosynthesis and pigment gland development (Table S4).

In the KEGG pathways analysis, the unigenes of the ‘blue’ module were significantly enriched into 16 pathways, which were prominently related to ‘metabolism of terpenoids and polyketids’, ‘metabolism of cytochrome P450’, ‘steroid hormone (isoprene-like) biosynthesis’, ‘metabolism’, ‘diterpenoid biosynthesis’, ‘lipid metabolism’, ‘sesquiterpenoid and triterpenoid biosynthesis’, ‘biosynthesis of various plant secondary metabolites’, and so on (Figure 6b and Table S5). Further, most of the hub genes were mainly enriched in the pathway of ‘metabolism of terpenoids and polyketides’, ‘cytochrome P450’, and ‘metabolism’. Note, in particular, six hub genes, *GH_A13G2336*, *GH_A03G0193*, *GH_D03G1778*, *GH_D13G2328*, *GH_A13G1576,* and *GH_D05G3845,* were significantly enriched in multiple metabolism pathways, such as ‘metabolism of terpenoids and polyketides’, ‘cytochrome P450’, ‘steroid hormone biosynthesis’, ‘diterpenoid biosynthesis’, and ‘sesquiterpenoid and triterpenoid biosynthesis’, which were directly involved in the gossypol biosynthesis and pigment gland development (Table S5). Obviously, the genes in the candidate module played important roles in the metabolic for the formation of gossypol biosynthesis and pigment gland in cotton.

### 3.6. Confirmation of DEGs by qRT-PCR

To validate the expression levels of DEGs identified by RNA-seq, 25 genes were selected to carry out qRT-PCR, including 18 common hub genes, 2 unique hubgenes for X18 which were the homologous genes of enzymes catalyzing the defined steps in MVA for gossypol pathways, 2 unique hubgenes for H1 which were the homologous gene of the enzyme in MVA pathways and the homologous gene of *pcC13-62* relating to the nectary formation of bean plant, and 3 non-hubgenes which were the homologous genes of *CYP76B*, *CGP1,* and *GoPGF* identified previously (Table S2).

**Figure 7:**
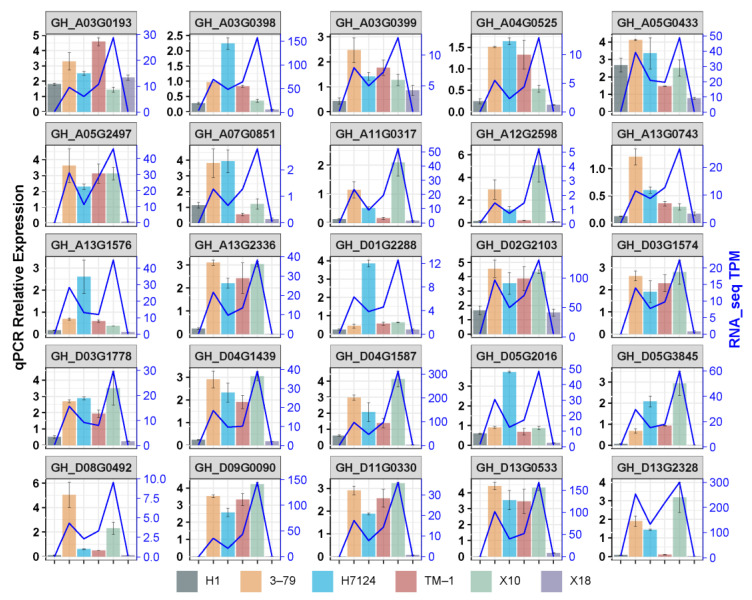
Validation of the DEGs by qRT-PCR.

As expected, most of the selected genes showed the similar trend of expression profiles between qRT-PCR and RNA-seq across samples, particularly the expression between glanded and glandless materials in each genetic background (*G. hirsutum* or *G. barbadense*) (Figure 7). The results confirmed the reliability of the RNA-seq data, which also confirmed that the reduction in or disappearance of gossypol and pigment glands were strongly associated with the down-regulation of genes identified in the candidate co-expression network.

## 4. Discussion

As the exclusive repository of toxic gossypol, pigment glands contribute to the resist of cotton against pathogens, pests, herbivores, and abiotic stresses [50,51,52]. However, the presence of toxic gossypol hinders the utilization of cottonseed rich in protein and oil. Worse still, the dephenolization of cottonseed is poor in operability, of a high cost, and contributes to environmental pollution. Obviously, it is valuable to breed new cotton cultivars with gossypol-free cottonseed and plants with a normal gossypol content, which can meet the multiple demands of agricultural production and broaden food resources for humans.

Usually, the number of glands can be used as a more convenient and efficient phenotypic indicator to study the gossypol content in cotton [8,9,10]. Numerous attempts have been made by breeders to study the morphogenesis of glands formation as well as the accumulation of related secondary metabolites in them [2,5,24]. However, the research progress of this area was at one time limited by the time-consuming and laborious task of map-based cloning for the corresponding loci. With the development of RNA sequencing, comparative transcriptome analysis was proved to be a rather straightforward and useful method to identify candidate genes that related to pigment gland formation [2,3]. In the present study, in addition to the identification of 13,113 DEGs by the comparative transcriptome analysis of several glanded and glandless cotton cultivars (Figure 2), a further WGCNA was innovatively applied to identify the key co-expression network and major genes for pigment gland formation (Figure 3, Figure 4 and Figure 5, Table S2).

Many of the DEGs in the candidate ‘blue’ module (network) were directly involved in gossypol biosynthesis and pigment gland development. Specifically, first, 24 and 7 genes were annotated as ‘CYP450 family’ and ‘terpene synthase/cyclases family’, respectively (Table S3), whose family members were participated in the metabolism of gossypol and/or pigment glands, such as the homologous genes of *CYP82D113* [5,53] *CYP76B6* [54], and *CDN*/*CDNC* ((+)-δ-cadinene synthase) [5,25,53] (Table S2). Those related genes were significantly enriched in the pathway of ‘metabolism of terpenoids and polyketides’, ‘metabolism of cytochrome P450, ‘Steroid hormone biosynthesis’, and ‘diterpenoid biosynthesis’, which enriched at least 48 genes of the candidate module. Second, *GH_D12G2619* and *GH_A12G2598*, as the homologous genes of *GoPGF*/*CGF3* which had been proved to control both gland morphogenesis and gossypol biosynthesis [23,24] through regulating the expression of *JAZ*, *WRKYs,* and *TPSs* [24], were enriched in the ‘anthocyanin compound metabolic (GO:0046283)’ and ‘flavonoid metabolic (GO:0009812)’, which enriched 8 and 13 genes of the candidate module, respectively. It should also be noted that *CGP1* (homologous gene of *GH_A07G0851*), a *MYB* transcription factor in the nucleus, can interact with *GoPGF* and form heterodimers to control the synthesis of gossypol and other secondary metabolites in cotton [3] (Table S3).

**Figure 8:**
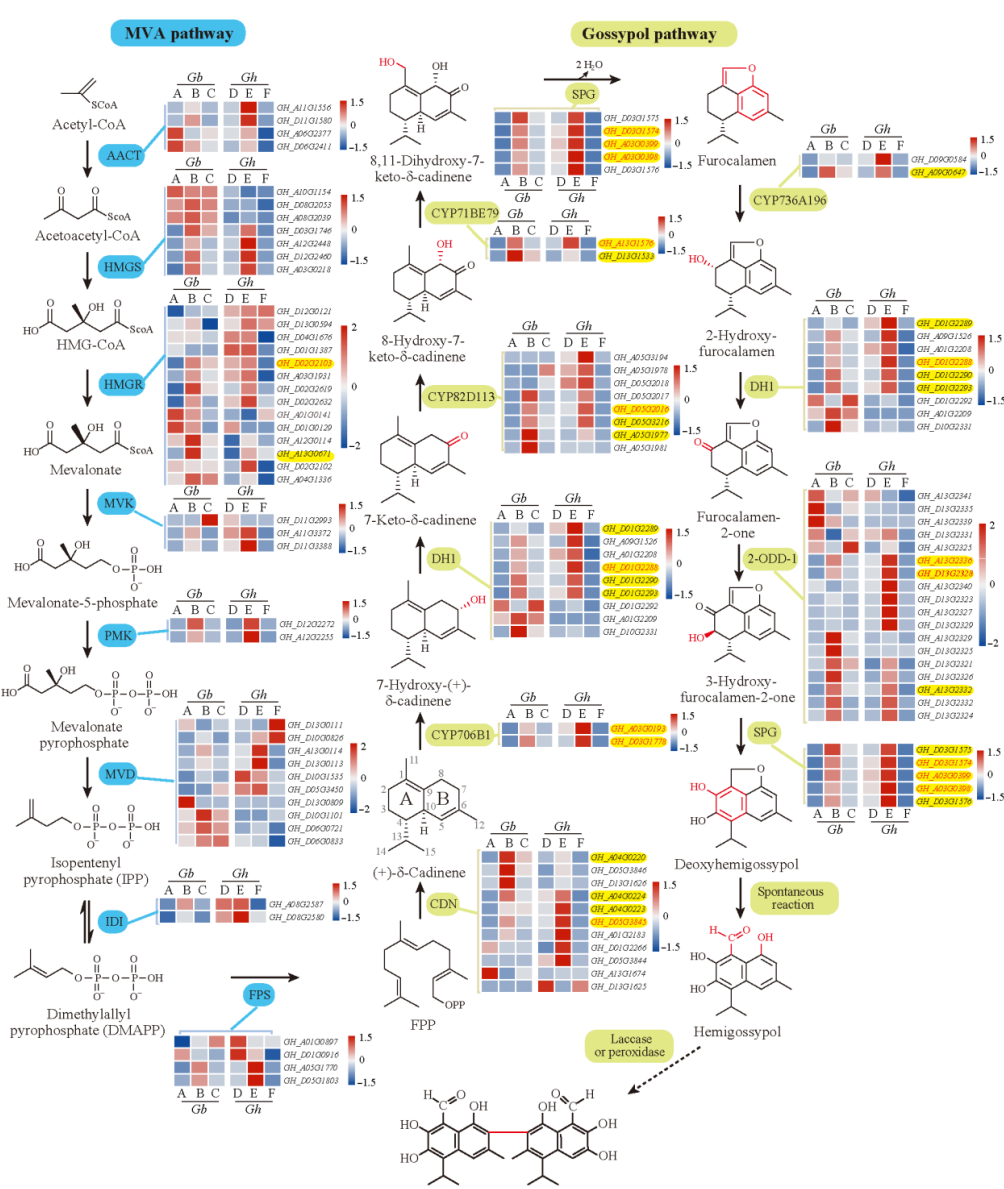
Genes of MVA pathway and gossypol pathway enzymes and their expression. Heatmaps indicate the relative expression levels of genes. The dashed arrow indicates an unidentified reaction. A–F above or under each heatmap indicate H1, 3–79, H7124, TM–1, X10, and X18, respectively. Abbreviations: 2-ODD, 2-oxoglutarate/Fe (II)-dependent dioxygenase; AACT, acetoacetyl-CoA thiolase; CDN, (+)-δ-cadinene synthase; DH, short-chain alcohol dehydrogenase; FPP, farnesyl diphosphate; FPS, farnesyl diphosphate synthase; HMGR, hydroxymethylglutaryl-CoA reductase; HMGS, hydroxymethylglutaryl-CoA synthase; IDI, isopentenyl diphosphate isomerase; MVA, mevalonate; MVD, mevalonate 5-diphosphate decarboxylase; MVK, mevalonate kinase; PMK, mevalonate 5-phosphate kinase; SPG, specialized glyoxalase I.

In addition, in our transcriptome results, 122 homologous genes with more or less expression in at least one cultivar were identified to be involved in the 18 enzymatic reactions of the know MVA and gossypol pathways [5,53], most of which were down-regulated in glandless materials (Figure 8). Additionally, full relative expressions of them in six cultivars were presented in Figure 8, serving as a reference resource for other relevant studies. Interestingly, lots of homologous genes of related enzymes were extensively expanded with tandem duplications (Figure 8), which appear to have arisen from local duplications, such as *CDN*, *DH1*, *CYP82D113,* and *2-ODD-1*. However, here, we are focused on genes that were effectively expressed in most samples (>50%), especially the DEGs clustered in the candidate ‘blue’ module. In total, in this module, 25 homologous genes of related enzymes were identified to be involved in at least 11 enzymatic reactions of the know MVA and gossypol pathways [53], especially each of the enzymatic reactions of the gossypol pathways. Additionally, all of them were significantly down-regulated or specifically not expressed in glandless materials (Figure 8, Table S2), which were very consistent with the results of previous studies [5,25,53,54]. Together, so many (42) homologous genes of known genes were clustered in the ‘blue’ module (or co-expression network) (Figure 5), which suggested that the WGCNA analysis provided a powerful approach to identifying candidate genes for pigment gland formation and gossypol biosynthesis and that the reliable scientific results were identified in present studies. Thus, we believe that the expression network constructed by 136 DEGs should play a crucial role in the formation of gland and gossypol in tetraploid cultivated cotton.

In total, 29 hub genes were highlighted in the co-expression network (Figure 5, Table S2), of which 13 were the homologous genes of know genes involved in the metabolism of pyruvate, terpenoids and polyketides, cytochrome P450, sesquiterpenoid, and triterpenoid biosynthesis. Notably, 12 hub genes were homologous to 8 enzyme genes of the know pathway mentioned above [53]. Specifically, two common hub genes *GH_A13G2336* and *GH_D13G2328* for *2-ODD-1*; two common hub genes *GH_A03G0193* and *GH_D03G1778* for *CYP706B1*; two common hub genes *GH_A03G0399* and *GH_D03G1574* and a unique hub gene *GH_A03G0398* for *SPG*; and common hub genes *GH_A13G1576*, *GH_D01G2288,* and *GH_D02G2103* for *CYP71BE79*, *DH1,* and *HMGR*, respectively. Moreover, a common hub gene *GH_D05G3845* was one of the homologous genes of *CDN*/*CDNC*, which was reported to decrease 95.1% of hemigossypolone and 96.7% of gossypol by VIGS [5]. *GH_D05G2016*, a unique hub gene for H1, was one of the homologous genes of *CYP82D113*, which was reported to decrease more than 50% of hemigossypolone and gossypol by VIGS [5]. Likewise, *GH_D08G0492*, a unique hub gene for H1, was detected as the homologous gene of *pcC13-62*, which was identified as a major nectar protein (nectarin) of the bean plant and is expressed exclusively in the stylopodium, where the nectary is located [55]. The relationship between *pcC13-62* and pigment gland or gossypol synthesis requires further study. Although identified as a non-hub gene in tetraploid cultivated *G. hirsutum* and *G. barbadense*, *GH_A04G0525* was detected as the homologous gene of *Gbi08G2110* (*CYP76B6*) regulated by *GoPGF*, which was identified in *G. bickii* and showed an important regulatory role for the biosynthesis of gossypol [54]. Altogether, in line with the crucial role of the identified gene network in the formation of gland and gossypol in tetraploid cultivated cotton, we believe that these hub genes should play an important role in the regulation of the expression network, which should be given priority in future studies.

Gossypol-free is a highly desirable trait for cottonseeds that increases the value of commercial cotton cultivars. Tissue-specific silencing is an effective measure to silence a gene in a particular tissue without affecting its expression in other tissues, and the trait created by this way is stable and heritable [56,57,58]. Therefore, the identification of candidate genes that are associated with gossypol biosynthesis or pigment gland formation provides us with the genetic resources which can be used for strict tissue-specific silencing to eliminate or reduce the gossypol in cottonseed kernel. Nowadays, as genetic engineering technology has developed, RNAi, CRISPR/Cas9, and CRISPR/Cas13a systems were applied for the destruction of specific transcripts [1,59,60]. Any such gene silencing technologies in conjunction with a seed-specific promoter can be used to eliminate the glands or gossypol from cottonseed only and develop a cotton cultivar with glanded plants and glandless cottonseed.

## 5. Conclusions

In the present study, 29 hub genes and related regulatory networks, which played key roles in the formation of gland and gossypol in tetraploid cultivated cotton, were identified by the RNA-seq of glanded and glandless cultivars distributed in *G. hirsutum* and *G. barbadense*. Our study provided the opportunity for a more accurate and comprehensive resolution of the genetic basis of gossypol and gland formation and should serve as a rich source for breeding cotton cultivars with gossypol-rich plants and gossypol-free cottonseed.

## Data Availability

The datasets generated during and/or analyzed during the current study are available from the corresponding author on reasonable request.

## Supplementary Materials

The following supporting information can be downloaded at: https://www.mdpi.com/article/10.3390/genes14061144/s1, Table S1: Summary of the RNA-seq data; Table S2: Gene information in blue module; Table S3: Function annotation of genes in blue module; Table S4: Go enrichment information in blue module; Table S5: KEGG enrichment information in blue module; Table S6: Primer sequences for qRT-PCR; Table S7: Expression TPM of top 20 significantly up-regulated and down-regulated genes.
